# Long-Range Correlations in Rectal Temperature Fluctuations of Healthy Infants during Maturation

**DOI:** 10.1371/journal.pone.0006431

**Published:** 2009-07-29

**Authors:** Georgette Stern, Julia Beel, Béla Suki, Mike Silverman, Jenny Westaway, Mateja Cernelc, David Baldwin, Urs Frey

**Affiliations:** 1 Division of Respiratory Medicine, Department of Pediatrics, Inselspital and University of Bern, Bern, Switzerland; 2 Department of Biomedical Engineering, Boston University, Boston, Massachusetts, United States of America; 3 Division of Child Health, University of Leicester, Leicester, United Kingdom; LMU University of Munich, Germany

## Abstract

**Background:**

Control of breathing, heart rate, and body temperature are interdependent in infants, where instabilities in thermoregulation can contribute to apneas or even life-threatening events. Identifying abnormalities in thermoregulation is particularly important in the first 6 months of life, where autonomic regulation undergoes critical development. Fluctuations in body temperature have been shown to be sensitive to maturational stage as well as system failure in critically ill patients. We thus aimed to investigate the existence of fractal-like long-range correlations, indicative of temperature control, in night time rectal temperature (T_rec_) patterns in maturing infants.

**Methodology/Principal Findings:**

We measured T_rec_ fluctuations in infants every 4 weeks from 4 to 20 weeks of age and before and after immunization. Long-range correlations in the temperature series were quantified by the correlation exponent, α using detrended fluctuation analysis. The effects of maturation, room temperature, and immunization on the strength of correlation were investigated. We found that T_rec_ fluctuations exhibit fractal long-range correlations with a mean (SD) α of 1.51 (0.11), indicating that T_rec_ is regulated in a highly correlated and hence deterministic manner. A significant increase in α with age from 1.42 (0.07) at 4 weeks to 1.58 (0.04) at 20 weeks reflects a change in long-range correlation behavior with maturation towards a smoother and more deterministic temperature regulation, potentially due to the decrease in surface area to body weight ratio in the maturing infant. α was not associated with mean room temperature or influenced by immunization

**Conclusions:**

This study shows that the quantification of long-range correlations using α derived from detrended fluctuation analysis is an observer-independent tool which can distinguish developmental stages of night time T_rec_ pattern in young infants, reflective of maturation of the autonomic system. Detrended fluctuation analysis may prove useful for characterizing thermoregulation in premature and other infants at risk for life-threatening events.

## Introduction

The identification of abnormalities in control of breathing, heart rate, and body temperature is clinically important in infants, particularly in the first 6 months of life where autonomic regulation and sleep undergo a critical maturational transition. These processes are interdependent in infants, where instabilities in thermoregulation and respiratory control, for example, may contribute to apnea and acute life-threatening events such as sudden infant death syndrome (SIDS) [1]–[6]. Specifically, Tappin et. al [7] found that there was a strong relationship between temperature regulation and control of breathing in infants during sleep, when life-threatening events often occur. Thus, there is an increasing interest in temperature control during maturation in infants with risk of SIDS [1], [4], [5], [8]. Establishing algorithms to quantify temperature control is also important in premature infants, in which temperature and breathing instabilities are frequent and often clinically difficult to quantify.

Temperature regulation is considered a favorable surrogate marker of maturation of autonomic control in infants since it is easy to measure and minimally invasive. Previous studies in infants during sleep have described the control and maturation of the regulatory system by observing the timing and magnitude of the night time fall in rectal temperature (T_rec_) [9]–[12]. However, using these criteria it is often difficult to distinguish the magnitude of the thermal fall from the normal fluctuation pattern in T_rec_. It is therefore important to identify new, automated techniques designed to reveal novel features of complex fluctuating signals. Recent work on physical and biological regulatory systems using a unifying and comprehensive approach based on system control theory has emphasized the non-linear nature of the feedback controls [13]–[15]. Such temporal dynamics in biological systems with a hierarchy of feedback loops often reveal fractal properties. Fractal-like long-range correlation properties in the fluctuations of physiological signals can be quantitatively described by using techniques such as the detrended fluctuation analysis (DFA) [16], [17] and provide important indicators of regulatory control in adults [17]–[23] and infants [24]–[26], the impact of disease on feedback control [15], [18], [22] and to the maturational stage of infantile breathing patterns [25]. However, while temperature regulation has been examined using such fractal analysis in adults [22], [23], it has not been applied in infants during maturation and with regards to the role of intrinsic and extrinsic influences on the thermoregulatory system. The influence of extrinsic perturbations [6], [24], [27], [28] is clinically relevant since it relates to the stability of feedback regulatory processes to transient perturbations. Perturbations can include environmental influences such as room temperature [6], [10], [27], [29]–[35] or immunization [36]–[40] that are known to both perturb temperature control in infants and be risk factors for SIDS.

The aim of this paper was to investigate whether night time T_rec_ patterns in infants exhibit fractal-like long-range correlations that can be characterized by DFA under clinically relevant natural conditions in their home environment. We assessed long-range correlations in time series of T_rec_ during sleep in the first 20 weeks of life and examined whether the correlation properties of T_rec_ change with maturation or can be influenced by external perturbations such as room temperature or by endogenous perturbations resulting from immunization.

## Materials and Methods

### Study design

We measured temperature fluctuations in 42 healthy term, unsedated infants during night time sleep at 4, 8, 12, 16, and 20 weeks of age. Additionally, temperature data were collected the night before and after immunization with diphtheria-tetanus-pertussis (DTP) and *Haemophilus influenzae* B (HIB) vaccines in 17 infants.

### Subjects

Healthy term infants born in the Leicester Maternity Hospital were recruited soon after birth. Infants with a history of perinatal asphyxia or major congenital disease were excluded from the study. Measurements were only performed if infants had been free of illness for 3 weeks.

### Ethics Statement

This protocol was approved by the Leicestershire Research Ethics Committee and performed with written informed parental consent.

### Temperature measurements

The temperature measurement setup has previously been described [31]. Briefly, night time temperature readings were collected using three probes; a soft rectal probe inserted 5 cm from the anal margin, a skin probe attached to the shin, and an ambient temperature thermometer in the bedroom attached to the measuring device at the bedside. In this study we report results from rectal and environmental recordings only. Measurements were performed in the home cots of the infants under conditions of room temperature and wrapping chosen freely by parents. Detailed recording of the wrapping and bed covers was not done as these are known not to affect T_rec_ pattern [6], [41]. The infants usually went to sleep after a feeding. All probes were connected to a Grant Squirrel Data Logger (Grant Instruments, Cambridgeshire, UK) set to record at one minute intervals. The minimum detectable temperature step was 0.1°C. Acquired data were downloaded to a computer for further analysis using a custom written analysis package for MATLAB (Mathworks, MA, USA).

### Data Analysis

All completed night time temperature time series with a continuous measurement period of at least 360 minutes were examined visually for signal quality. Datasets with technical problems or artifacts such as probe loss (defined as drop of temperature under 36°C as well as visual evidence of probe loss in temperature signal) were excluded from further analysis.

### Classical method of temperature control assessment in infants

Longitudinal night time temperature pattern studies are reported to show an abrupt change in healthy young infants at around 7–16 weeks of age to a mature temperature pattern which persists into adulthood [9], [11]. A maturational change in thermal control to an adult-like pattern has been characterized by a sudden drop in T_rec_ from more than 37°C during wakefulness to a minimum of 36.4°C within 2 hours of bedtime, after which it remains low for a few hours before increasing to around 37°C before waking. The change from an immature (infant-like) to a mature (adult-like) temperature pattern is maintained across a wide range of parental practices of room heating, clothing and wrapping indicating a considerable capacity for thermoregulation [6], [41].

### Detrended fluctuation analysis (DFA)

To estimate the long-range correlation properties in night time T_rec_ fluctuations, we looked at minute-to-minute T_rec_ time series with DFA as introduced by Peng et al. [17], [20]. The DFA is a technique that quantifies the self-similar scaling and correlation properties of non-stationary time series, reflecting intrinsic fractality. This method was introduced for detection of intrinsic correlation properties of complex physiological signals while simultaneously avoiding the detection of false correlations due to the non-stationary nature of time series. It estimates the fluctuation function of a time series using Equation 1;
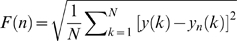
(1)where *F(n)* is the root-mean-square fluctuation of the original signal and *N* is the length of the data record. To calculate *F(n)* of the minute-to-minute time series of the rectal temperature *T_rec_(k)* containing *N* data points, first the mean T_rec_ was subtracted from *T_rec_(k)* and integrated. The integrated function *y(k)* was then divided into non-overlapping windows of equal length *n* and a linear regression *y_n_(k)* was fit in each window, representing the local trends for each window size *n*. The time series was then detrended by subtracting the local trend in each window. This process is then repeated for increasing *n*. Typically *F(n)* will increase with window size *n*. If this increase is linear on a log-log plot, then *F(n)* is said to follow a power law functional form *F(n) = A n^α^*, where α is the correlation exponent and *A* is the amplitude of the power law fluctuation function. Thus, if *F(n)* is a power law, the self-similarity of fluctuations in the original signal can simply be characterized by a single parameter, α, the slope of the log-log plot.

An uncorrelated, random process such as white noise has an α = 0.5. Increasing α implies increasing the strength of long-range correlations with the signal being smoother and more deterministic. The special case of α = 1 corresponds to 1/f noise while Brownian noise, the integration of white noise, yields an α of 1.5.

Additionally, the calculation of α is sensitive to the length of the recording so that in short time series, the calculated α can be substantially different from the true value. Scaling exponents obtained from data records with *N*<256 can have relatively large uncertainty in the estimation of α due to finite size effects [25]. Therefore, the inclusion criteria of time-series of at least 360 data points and for which the correlation coefficient of the regression line between *n* and *F(n)* was r^2^>0.97 was sufficient to minimize error and ensure quality of the DFA method.

### Statistical Analysis

#### Long-range correlations and repeatability

To verify the existence of long-range correlations, α was calculated on the T_rec_ time series of all technically acceptable night time sets of temperature measurements using DFA. The series were then randomized to remove the original ordering and re-analyzed with DFA. In the latter case, the time series should take on the properties of an uncorrelated random process with α = 0.5. To test the repeatability of the DFA method, α of the night time T_rec_ time series in 8 infants were collected on adjacent nights.

#### Changes with maturation

The final dataset was comprised of a mixture of cross-sectional and longitudinal data. To account for this, we used three different statistical approaches to show the influence of maturation on temperature control: i) We examined longitudinal datasets in a subgroup of 23 infants, for whom measurements were available at 4 or 8 weeks and 12, 16, or 20 weeks by paired t-test. ii) After ensuring a normal distribution of α, linear regressions through the cross-sectional and longitudinal datasets were performed using a random-effects GLS regression model with infant-specific intercepts adjusted for sex and weight at the measurement. With this model, the relationship between α and age can be assessed by taking into account the individual associations. iii) Lastly, to make our data comparable to published studies in infants, the maturational stage of a temperature pattern was determined to have either an immature pattern, a mature pattern, or an indeterminable pattern [9], [32]. The correlations exponents α in the immature and mature groups were compared using a random-effects GLS regression model.

#### Concomitant factors

In order to determine the effect of room temperature on intrinsic temperature regulation, median room temperature in a subgroup of 57 infants in which room temperatures were nearly constant over the observation period (expressed by its skewness and lack of major fluctuations) was added as an exposure into the multivariate models of the association between mean T_rec_ and α with age in infants at 4, 8, and 12 weeks using the random-effects GLS regression model. The effect of immunization on T_rec_ and α was investigated in 17 paired T_rec_ series recordings recorded during the night prior to and immediately after immunization by paired t-test.

#### Sensitivity and robustness analysis

In those infants with a clear maturation stage could be defined using the classical definition, we examined the possibility that trends in the data as well as the quantization of the time series due to the resolution of the temperature probe would affect the DFA calculation. A squared term in the quadratic equation fit to the temperature series, directly related to the initial temperature drop of the time series, was added to the linear regression model relating α and age. Similarly, due to the limited resolution of the temperature probe, infants with an immature temperature pattern have fewer possible values in the digitized series compared to mature infants. Therefore we investigated the difference in α due to digitizing a simulated Brownian noise to 8 and 12 discrete steps.

## Results

We aimed to measure night time T_rec_ in 42 recruited infants every 4 weeks from 4 to 20 weeks of age. Due to exclusion of technically unacceptable datasets, the final dataset was comprised of 95 time series that were acceptable and included a mixture of cross-sectional and longitudinal data (Table 1). One infant had acceptable data from all 5 stages, while 7 infants had acceptable data from 4 stages, 10 on 3 stages, 8 on 2 stages, and 16 infants had technically acceptable data from only one measured occasion.

**Table 1:** Infant characteristics and changes in rectal temperature regulation with maturation, room temperature, and immunization.

	Category	Group Size	Age (days)	Sex (male)	Weight (g)	T_rec_ (°C)	Recording Time (min)	α	Room Temp (°C)
			mean (SD)	n (%)	mean (SD)	mean (SD)	mean (SD)	mean (SD)	median (range)
Included Infants	All	95	64.5* (132)	44 (46)	5450 (5400)*	36.9* (1.1)	651 (168)	1.51 (0.11)	
Maturation	4weeks	25	31* (12)	11 (44)	4500 (555)	37.0 (0.2)	655 (177)	1.42 (0.07)	
	8weeks	28	61 (4)	13 (46)	5320 (650)	36.9* (0.6)	627 (174)	1.49 (0.08)	
	12weeks	23	87* (19)	11 (48)	6020 (760)	36.7 (0.2)	674 (188)	1.55 (0.11)	
	16weeks	11	115* (19)	5 (45)	6500 (730)	36.7 (0.3)	677 (142)	1.60 (0.13)	
	20weeks	8	145 (7)	4 (50)	7730 (1010)	36.6 (0.2)	622 (108)	1.58 (0.04)	
	Immature	13	46 (26)	9 (70)	5310 (1090)	37.1 (0.15)	723 (141)	1.42 (0.06)	
	Mature	18	106 (28)	8 (44)	6140 (950)	36.7 (0.13)	614 (155)	1.59 (0.08)	
Room Temperature	4weeks	16	31* (12)			36.9* (0.78)	631 (190)	1.43 (0.08)	21.6 (6.41)
	8weeks	23	61 (4)			36.9* (0.6)	624 (175)	1.48 (0.08)	21.0 (4.74)
	12weeks	18	87* (19)			36.7 (0.2)	658 (171)	1.57 (0.11)	20.0 (4.45)
Immunization	before immunization	17	63* (0.47)			36.8 (0.2)	618 (156)	1.49 (0.12)	
	after immunization	17	64* (0.53)			37.1 (0.3)	635 (178)	1.45 (0.13)	

* Data not normally distributed described by median (range).

### Long-range correlations and repeatability

**Figure 1:**
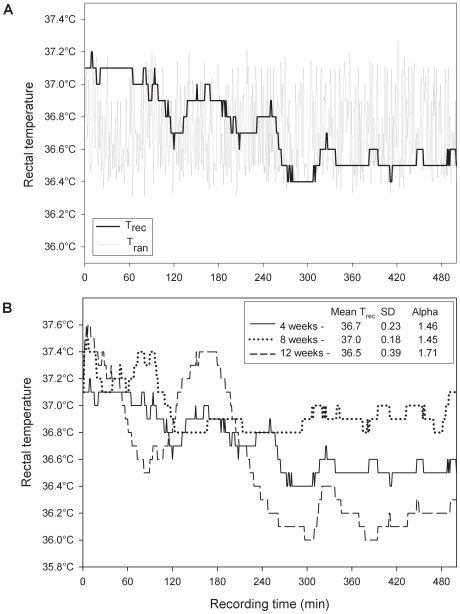
Night time T_rec_ time series. a) Night time rectal temperature (T_rec_) recorded over 508 minute intervals from a single infant with a mean (SD) of 37.7°C (0.2). The corresponding random temperature (T_ran_) was generated by shuffling data points of T_rec_. b) Longitudinal night time rectal temperature traces T_rec_ from the same infant as Figure 1a at 4, 8, and 12 weeks with mean and standard deviation of T_rec_ and corresponding α shown.

**Figure 2:**
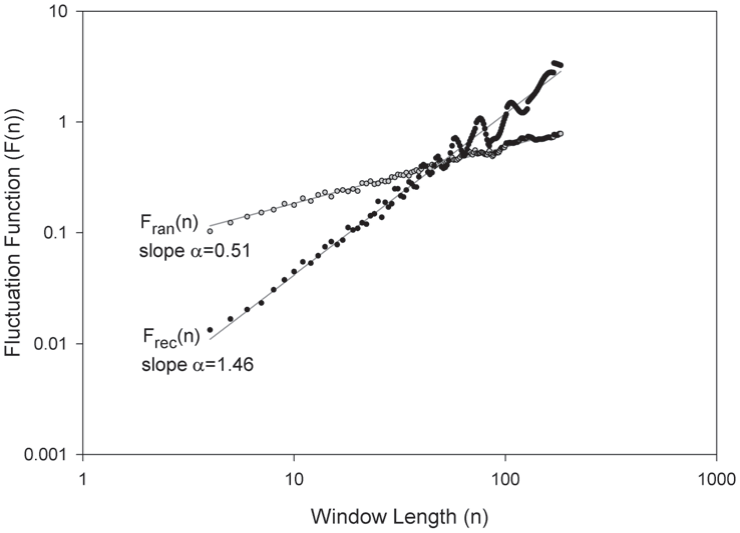
Fluctuation function *F(n)* of T_rec_ and T_ran_. Root-mean square fluctuation function *F(n)* of T_rec_ and T_ran_ from Figure 1a shown as *F_rec_(n)* and *F_ran_(n)* respectfully on a log-log plot and their regression line fit over window sizes n up to 180 minutes with slopes α_rec_ = 1.46 and α_ran_ = 0.51 respectfully.

An example of a night time T_rec_ recording as a function of time from a single subject and its randomized time series is shown in Figure 1a. The longitudinal night time T_rec_ recordings from the same subject at 4, 8, and 12 weeks are shown in Figure 1b. A plot of the corresponding fluctuation function *F(n)* of the time-series in Figure 1a can be seen in Figure 2 on a log-log plot versus window size *n*. In 88 of the 95 datasets, *F(n)* had a linear trend up to window sizes of at least 180 minutes that could be characterized by the slope α of the linear regression with an r^2^>0.97 (Table 1). In 7 of 95 recordings the linear regression had an r^2^<0.97 and a smaller window range was chosen to fit a linear regression with r^2^>0.97. Overall mean (SD) of α for the 95 T_rec_ night time time series was 1.51 (0.11). When the data were randomized, the average slope of the fluctuation function became 0.51, indicative of a random process. In our time series data we found no evidence of crossover phenomena implying that α did not change for different time scales (ie. window size, *n*) [25].

The mean α collected on adjacent nights for the 8 infants to test the repeatability of the method was 1.51 (0.11) on the first night (α_1_) and 1.51 (0.11) on the second night (α_2_) with a mean difference of −0.005 (95% CI −0.081 to 0.076) that was not statistically significant.

### Changes with maturation

**Figure 3:**
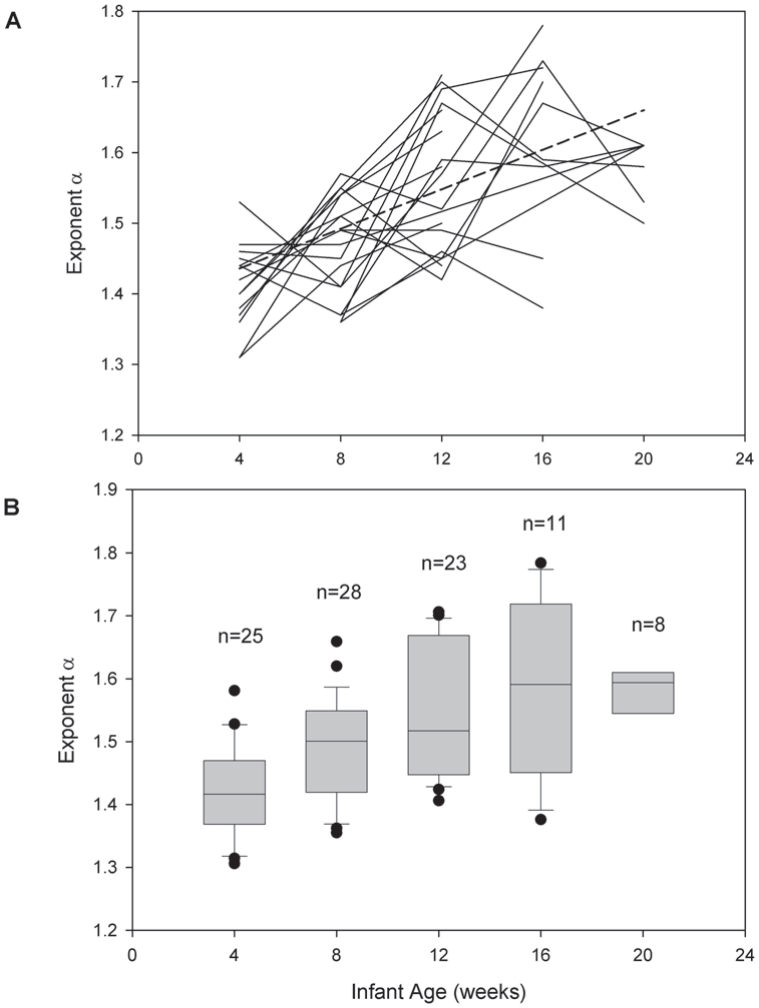
Change in α with age. a) Longitudinal measurements in infants with at least 3 measurements (solid lines). One infant had acceptable data from all 5 stages, 7 infants had acceptable data from 4 stages, and 10 from 3 stages. There is individual variability in the change in α with age, but there is an overall increase in α of 0.012 per week of age (95%CI 0.005 to 0.019, p = 0.001) (dotted line). b) A box plot of α in all included infants grouped by age at 4, 8, 12, 16, and 20 weeks. The boundary of the box closest to zero indicates the 25^th^ percentile, the line within the box marks the median, the black dots are the outliers, and the boundary of the box farthest from zero indicates the 75^th^ percentile. Error bars above and below the box indicate the 90^th^ and 10^th^ percentiles.

The scaling exponent α was normally distributed in all age groups (Table 1). α calculated from temperature time series of subjects which had at least three longitudinal measurements (n = 18) can be seen in Figure 3a. A box plot of α for all of the included infants stratified by age group is shown in Figure 3b. There was a small significant drop in mean T_rec_ between the age groups starting at around 12 weeks of −0.021°C per week of age (95%CI −0.028 to −0.013, p<0.001). All three methods of analyzing the age effect on α showed a significant increase of α with age. Longitudinal data in a subgroup of 23 infants showed that α values at 4–8 weeks (mean (SD) 1.44 (0.08)) were significantly lower than those at 12–20 weeks (1.58 (0.10), p<0.001). Regression analysis of all 95 time series using random-effects model showed a significant increase in long-range correlations with age of α = 0.012 per week of age (95%CI 0.005 to 0.019, p = 0.001), adjusted for weight and sex of the infant. If only infants with 3 or more longitudinal measurements were included in the regression, there was a slightly higher increase in alpha with age of α = 0.015 per week (95%CI 0.007 to 0.024, p<0.001). Of the 95 acceptable datasets, 31 of these were determined to have an immature (n = 13) or mature (n = 18) night time T_rec_ patterns (Table 1). An indeterminate group of 61 datasets in which a clear pattern was not identifiable was excluded. Of those with immature or mature night time temperature patterns, it was found that there was a mean increase in α of 0.17 in the mature group compared with the immature group, (95%CI 0.12 to 0.22, p<0.001).

### Concomitant factors

Night time room temperatures were mostly constant in 57 of the 95 measurements (Table 1). The relationship between mean room temperature and mean T_rec_ was not significant (p = 0.239). The addition of mean room temperature into the random-effects model did not affect the relationship between α and age, even when stratified by age group.

While there was a significant increase in the median T_rec_ after immunization in the 17 infants of 0.28°C (95%CI 0.17 to 0.40, p<0.01), there was no significant change in α (p = 0.49) (Table 1).

### Sensitivity Analysis

The drop in the T_rec_ temperature time series shortly after the onset of sleep was fit with a quadratic curve in infants in the immature/mature subgroups. The squared term in the temperature curve (group mean (SD)) for the immature group was 2.90×10^−6^ (1.67×10^−6^) and for the mature group was 6.04×10^−6^ (3.67×10^−6^). The relationship between α and age was not affected by the addition of the squared term into the random-effects model.

When DFA was run on a simulated Brownian noise signal quantized to 8 and 12 discrete steps, α was significantly decreased from 1.5 to around 1.30, but there was only a minor difference between the calculated α sampled at 8 or 12 steps. Quantization of a simulated a time series with an α of 1.75 lead to an estimation of α around 1.5, implying that due to the limited resolution of the temperature probe, we underestimate the actual correlation exponent of long-range correlations in T_rec_ fluctuations in this study.

## Discussion

For the first time, using DFA, this study provides evidence that temperature regulation in healthy infants exhibits fractal-like long-range correlations with an average correlation exponent α of 1.5, indicating that temperature control in infants is highly correlated and hence deterministic. The strength of long-range correlations increased significantly during the first 5 months of life from 1.42±0.07 at 4 weeks to 1.58±0.04 at 20 weeks, a period over which known developmental changes of autonomic control in infants occur and the risk for SIDS is the highest. The increase in α is consistent with increasing determinism in temperature control with age. Furthermore, α was found to be stable against room temperature and immunization, two factors which have been widely discussed in the literature as potential risk factors for SIDS. Therefore, α has the potential to be an observer-independent parameter to quantify the maturational stage of temperature control, representative of other autonomic control systems, in infants at risk for SIDS or premature infants.

### Temperature control in infants

Thermoregulation involves a balance between endogenous thermogenesis and heat loss by peripheral vasoregulation. Heat production depends on the metabolism of the infant, including muscular activity, non-shivering thermogenesis, and fever. Heat loss is determined by sweating, peripheral vasodilatation of the skin, insulation, ambient temperature, radiation to and from heat sources, and convection [30], [35]. Multiple central controllers with separate and independent functions are thought to maintain thermo-neutrality in newborns [8]. Compared to adults, newborns are characterized by a high skin surface area to body mass ratio, enhancing heat losses to the environment in the form of convection, evaporation and radiation [8]. As a consequence, in a cold environment, the newborn can rapidly become hypothermic. Contrary to neonates, the thermal balance of the 3-month-old infant is shifted in favor of heat conservation. This can be explained by growth, the increase in metabolic rate with age, and the decrease in the surface area to body mass ratio associated with the thicker layer of subcutaneous fat. The 3-month-old must dissipate 50% more heat per unit body surface area than during the first weeks of life and consequently, is particularly vulnerable to hyperthermia [8]. Our finding that there is an increase in intrinsic long-range correlations in the first months of life suggests generally smoother fluctuations in temperature which might be due to the body's change in thermocapacity.

### Long-range correlations in night time infant rectal temperatures

If a process is completely random (e.g. white noise), the output values are uncorrelated and the corresponding α is 0.5. If α>0.5 then there must be some physical, physiological or biological mechanisms that act to influence the future values of the output. Although measured with different devices and different resolutions, temperature regulation in infants is highly deterministic with higher values of α (∼1.5) compared to breathing regulation (α∼0.7–0.8) [25] in infants or heart rate control (α∼1.0) [17] in adults.

Long-range correlations in body surface temperature have been described in healthy adults [23]. We found α to be higher in infants in comparison to adults. This may be related to the fact that we measured rectal temperature rather than skin temperature, where the latter is probably less deterministic due to environmental heat loss.

### Changes in α with maturation

We found a significant increase in α with increasing age and maturation by comparing a subset of longitudinal data as well as by fitting a random-effects regression through the longitudinal and cross-sectional data taking into account the different associations on an individual level. Thus, the thermoregulatory system in infants becomes increasingly deterministic and less variable with increasing age until a mature stage, despite the fact that mean core temperature only slightly decreases. This may represent an improving functional maturity of the temperature control feedback loop against internal and external perturbations. This finding appears to support the interpretation that the thermal mass of infants increases dramatically and the surface area relative to mass falls in the first 6 months of life.

Furthermore, if α changes from one value to another, then either the inputs to the control system or some of the mechanisms involved in the control system must also undergo changes. Since external perturbations did not affect α, it is reasonable to assume that it is the control system itself that undergoes changes during maturation. As a consequence, the correlated fluctuations observed in T_rec_ are representative of the properties of the various mechanisms that regulate T_rec_. Therefore, a change in α implies that some of the regulatory mechanisms contributing to the correlations must also be altered. It is in this sense that we use the term “regulation”.

While in adults an increase in α, or an increase in determinism and smoothness, has been linked to aging and disease [15], [18], [22], [23], we speculate that this is not the case in the maturing infantile healthy temperature control. During infant maturation, an increase in α signifies less variability and more stability in the control system, whereas in aging and disease, a too high α can perhaps be associated with less adaptability of the system. For example, a periodic signal represents a completely deterministic system with very little tolerance for change. Perhaps there exists a tradeoff between too little and too much variability or determinism [42].

The classical way to assess temperature control by measuring night time mean T_rec_ and magnitude of T_rec_ drop at the onset of sleep is observer-dependent and was only possible in one-third of the temperature series whereas the automated DFA analysis is robust and easy and could be of clinical use. Previous papers [9], [12], [34], [41] suggest that temperature maturation changes abruptly from an immature to a mature adult-like control, however they suggest that this may happen at different ages in the individuals.

### Effect of room temperature

Although in premature infants it is well known that room temperature influences body temperature [35], term infants are known to maintain a remarkably stable body temperature. Changes in environmental conditions may disturb thermoregulation, resulting in possible disruption of other regulatory processes, such as respiratory control [1], [27], and contributing to adverse clinical events, such as SIDS [5]. However, epidemiological evidence suggests that there is no correlation between room temperature and SIDS [43]. We found no correlation between mean room temperature and mean T_rec_ or α, irrespective of age, supporting the notion that term infants are able to maintain a stable body temperature within a reasonable range despite changes in room temperature.

### Effect of immunization

Immunization frequently produces a transient febrile episode [37], [38]. We did not find any association between immunization and α, despite a small rise in mean T_rec_ during sleep. This contrasts with the situation during maturation, where T_rec_ slightly decreases with age while long range correlations characterized by α increase. This suggests that while maturation appears to alter thermoregulation, no real change in the long-term control of temperature occurs after the short-term perturbation of immunization. It is possible that immunization simply introduces an offset in the temperature fluctuations without affecting the regulatory process itself. Our results are consistent with the notion that immunization changes the temperature set point, but not the correlation properties of the relative fluctuations.

### Limitations of the method

There are several potential technical factors that could compromise the estimation of the correlation exponent α using the DFA method. One is the larger temperature drop during the night in mature infants. The other is the limited resolution of the temperature probe. Based on computer simulations, we found that while these factors could influence the value of α, they did not eliminate the relationship between α and age. A measurement resolution of 0.1°C is standard and widely used [7], [23], and is sensible in terms of temperature signal to noise ratio. In our measurements, we found a value of α of ∼1.5. We cannot say what the α would be if signal had an infinite resolution, but computer simulations suggest that we are underestimating the true value of α. However, the magnitude of the effect of the number of temperature steps was found to be small compared to the relationship between α and age.

There are several explanations for the incomplete nature of our longitudinal dataset. It was often difficult to motivate parents to have their infant's T_rec_ recorded on several occasions. Also, since the infants were measured at home throughout the night, there were instances of probe loss due to defecation and mothers were instructed not to replace the probe if lost to prevent injury of the child. This could have also occurred more in the older infants due to the increased motility with age. These measurements were excluded from the study. Consequently, we were able to collect 95 partially longitudinal datasets out of a potential 210 recordings from 42 infants. However, the mixed (95 datasets), longitudinal (23 subjects) and cross sectional data (42 subjects) all showed a significant increase in α with age until a mature stage.

During measurement periods of up to 6 hours, infants undergo several changes in sleep stages [44]. Mean body temperature in newborns is not related to sleep stage [6], [29], [45], whereas the mature temperature pattern in older infants are dependent on bedtime [11]. It is unlikely that this has a significant influence on α since this parameter characterizes the whole data series consisting of at least 360 minutes during which time several sleep cycles can occur. We did not record sleep stages, and therefore it is unclear whether α varies between sleep stages, but it has been reported that fractal properties of infant breathing are independent of sleep stage [26].

### Summary and Implications

We found that temperature regulation in infants exhibits fractal-like long-range correlations as determined by DFA with an exponent α of around 1.5. The value of α increased with age while remaining robust to the influences of immunization and environmental temperature within the normal range. This increase in control at around 3 months could be due to the increased heat capacity of older infants. Such a high value of α suggests more a deterministic and smooth thermoregulation in mature infants.

Several situations exist where the autonomous control of temperature is thought to be immature or altered including prematurity, SIDS, old age, critical illness or after medication with certain drugs [46]. In such circumstances, a stable baseline core body temperature may not be maintained when exposed to environmental temperature fluctuations [8], [47]. Clinically it is often difficult to determine the exact maturational stage of the thermoregulatory control system in infants. A simple, observer-independent, and robust measure of temperature regulation such as α may be clinically helpful in future studies and to identify infants at risk.
